# Single-cell RNA sequencing reveals regulation of fetal ovary development in the monkey (*Macaca fascicularis*)

**DOI:** 10.1038/s41421-020-00219-0

**Published:** 2020-12-29

**Authors:** Zheng-Hui Zhao, Chun-Yang Li, Tie-Gang Meng, Yan Wang, Wen-Bo Liu, Ang Li, Yi-Jun Cai, Yi Hou, Heide Schatten, Zhen-Bo Wang, Qing-Yuan Sun, Qiang Sun

**Affiliations:** 1 State Key Laboratory of Stem Cell and Reproductive Biology, Institute of Zoology, Chinese Academy of Sciences, 100101 Beijing, China; 2 University of Chinese Academy of Sciences, 100049 Beijing, China; 3 Institute of Neuroscience, CAS Center for Excellence in Brain Science and Intelligence Technology, State Key Laboratory of Neuroscience, CAS Key Laboratory of Primate Neurobiology, Chinese Academy of Sciences, 200031 Shanghai, China; 4 Fertility Preservation Lab, Reproductive Medicine Center, Guangdong Second Provincial General Hospital, 510317 Guangzhou, Guangdong China; 5 Department of Reproductive Medicine Center, Third Affiliated Hospital of Guangzhou Medical University, 510150 Guangzhou, Guangdong China; 6 Department of Veterinary Pathobiology, University of Missouri, Columbia, MO 65211 USA

**Keywords:** Cell growth, Bioinformatics

## Abstract

Germ cells are vital for reproduction and heredity. However, the mechanisms underlying female germ cell development in primates, especially in late embryonic stages, remain elusive. Here, we performed single-cell RNA sequencing of 12,471 cells from whole fetal ovaries, and explored the communications between germ cells and niche cells. We depicted the two waves of oogenesis at single-cell resolution and demonstrated that progenitor theca cells exhibit similar characteristics to Leydig cells in fetal monkey ovaries. Notably, we found that *ZGLP1* displays differentially expressed patterns between mouse and monkey, which is not overlapped with *NANOG* in monkey germ cells, suggesting its role in meiosis entry but not in activating oogenic program in primates. Furthermore, the majority of germ cell clusters that sharply express *PRDM9* and *SPO11* might undergo apoptosis after cyst breakdown, leading to germ cell attrition. Overall, our work provides new insights into the molecular and cellular basis of primate fetal ovary development at single-cell resolution.

## Introduction

Fetal ovary development is a well-orchestrated complex process that involves the transitions of multiple cell states and communications between germ cells and niche cells. In primates, female germ cell (FGC) development processes at embryonic stages mainly include oogonia proliferation, meiosis initiation, germ cell attrition, and primordial follicle formation, which are accompanied with dynamic chromatin repackaging and transcriptional regulation. Moreover, FGCs initiate meiosis asynchronously in a wave from anterior to posterior, which results in the heterogeneity of germ cell populations in a fetal ovary^1^. Somatic progenitor cells in female gonads will adopt the ovary-specific cell fate after sex determination, which leads to their differentiation as granulosa cells in supporting cell populations or as theca cells in steroidogenic cell populations^2^. Interestingly, the FGC fate mainly relies on the ovarian environments established by somatic cells rather than the sex chromosomes in germ cells^3^.

Despite the mass data produced in recent years on fetal ovary development^4–6^, several important questions regarding the key developmental events, such as two waves of oogenesis, FGC attrition, and primordial follicle formation, have not been fully demonstrated, especially in non-human primates. In addition, although the origins of germ cells and granulosa cells are well established^2,7^, the origin of theca cells in primates remains unknown. Furthermore, the germline–niche communications through signaling pathways for critical events during fetal ovary development are poorly integrated.

Single-cell RNA sequencing (scRNA-seq) approach can efficiently identify cell types, uncover heterogeneity, and construct developmental trajectories, which is well suited for exploring fetal ovary development. To further improve our understanding on the germ cell development and somatic cell differentiation, we here performed scRNA-seq of fetal monkey ovarian cells, especially in late embryonic stages, through 10× Genomics Chromium platform. And a transcriptional cell atlas of all cell types in the fetal ovary was established.

## Results

### Identification of the ovarian cell types using single-cell transcriptomes

**Fig. 1:**
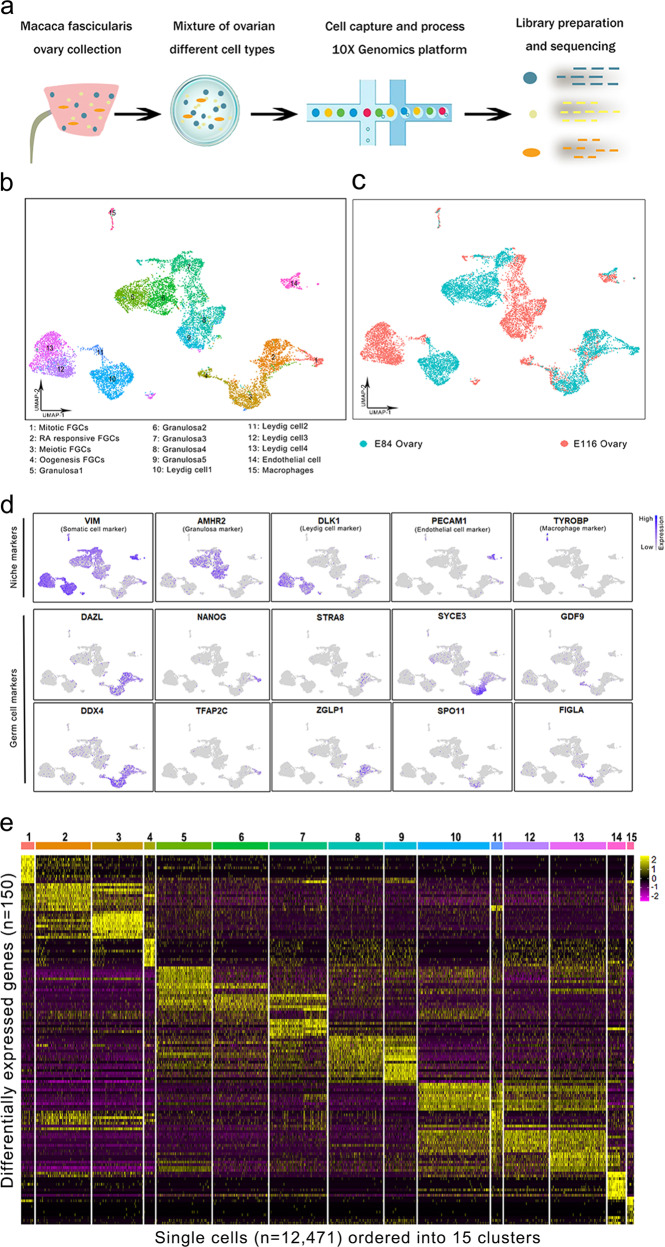
Single-cell transcriptome profiling of fetal monkey ovaries. **a** Schematic diagram of the experimental design. In total, fetal ovaries were collected from E84 and E116 monkey fetus and further enzymatically digested into single-cell suspension, which were then captured using 10× Genomics technology. The libraries of single-cell transcriptome were generated using the single-cell 3′ reagent V3 kits according to the manufacturer’s protocol. **b** UMAP and clustering analysis of single-cell transcriptome data from fetal monkey ovaries. The 15 clusters were assigned and colored as indicated on the figure key. **c** UMAP plot of single-cell transcriptome data with cells colored based on their embryonic stages. **d** Expression patterns of selected markers for the identification of cell clusters. Blue indicates high expression and gray indicates low or no expression, as shown on the figure key. **e** Heatmap showing top 10 differentially expressed genes across 15 cell clusters, and 12,471 single cells were visualized.

Single-cell suspensions of fetal Macaca fascicularis ovaries were individually captured and processed with the 10× Chromium system (Fig. 1a). Sequencing data from two scRNA-seq libraries derived from the two stages of ovary samples were integrated for processing and analysis. From a total of 21,036 cells, we obtained an average of 57,269 reads per cell and 1593 genes per cell (Supplementary Fig. S1a). Following quality control, 12,471 cells were retained for further analysis.

To classify the different cell populations in fetal monkey ovaries, we performed unsupervised graph clustering through Seurat package^8^ to group the cells into cell clusters based on the similarities in their transcriptome profiles. Overall, the 15 transcriptionally distinct clusters were classified and visualized using UMAP (Uniform Manifold Approximation and Projection) (Fig. 1b)^9^. Notably, the clusters of germ cells (1, 2, 3, 4), endothelial cells (14), and macrophages (15) exhibit obvious overlap between E84 and E116 ovaries, while the granulosa cell populations (5, 6, 7, 8, 9) exhibit slight overlap between E84 and E116 ovaries, and many cells derived from E84 and E116 ovaries were grouped into cluster 7. In addition, the Leydig cells (10, 11, 12, 13) exhibit no overlap between E84 and E116 ovaries (Fig. 1c). The granulosa cells and Leydig cells from E84 ovary and E116 ovary are separated into different clusters, which suggests that these cells may undergo dramatic differentiation during the development from E84 to E116 ovary. To further identify the clusters, we assigned the clusters based on known cell-type marker genes (Fig. 1d). The germ cell-specific marker genes were expressed solely in clusters 1–4 (*DAZL*, *DDX4*). The clusters 5–15 are somatic cell populations (*VIM*), which include granulosa cells (*AMHR2*), Leydig cells (*DLK1*), endothelial cells (*PECAM1*), and macrophages (*TYROBP*). Also, the germ cell marker genes displayed developmental stage-specific expression patterns. For instance, primordial germ cell marker gene *TFAP2C* and pluripotency marker gene *NANOG* were expressed in the early mitotic FGCs, whereas *STRA8* was expressed in retinoid acid (RA) signaling-responsive FGCs. The cells in cluster 3 were meiotic prophase FGCs and specifically expressed *SYCE3* and *SPO11*. Finally, the cells in cluster 4 were oogenesis phase FGCs and clearly expressed *GDF9* and *FIGLA* (Fig. 1d). Collectively, the sequential expressed germ cell markers in clusters 1–4, respectively, could mirror the temporal order of early FGC development. Interestingly, our results also demonstrated multiple transcriptionally distinct subpopulations within the granulosa cells (five clusters) and Leydig cells (four clusters). Additionally, the top 10 differentially expressed genes were selected across cell clusters for the identification of each cell type (Fig. 1e). Together, we have profiled the transcriptomes of all cell types in the fetal monkey ovary in the present study.

### Cell lineage reconstruction reveals two waves of oogenesis

**Fig. 2:**
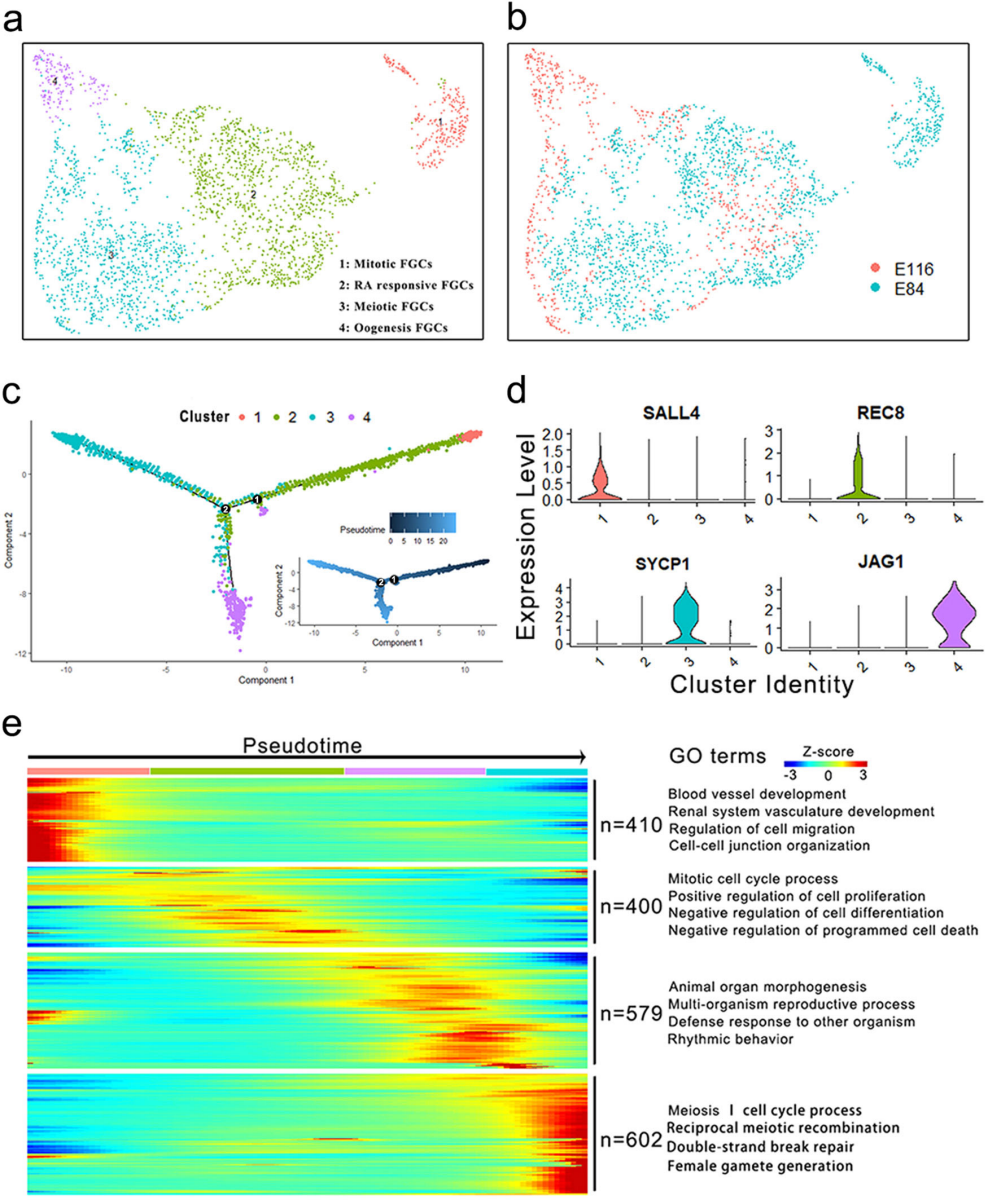
Cell lineage reconstruction and identification of the germ cells. **a** Isolation and re-clustering of the germ cell populations at a higher resolution. The assigned clusters are consistent with that in Fig. 1b. **b** UMAP plot of single-cell transcriptome data with germ cells colored based on their embryonic stages. **c** Pseudotime analysis of germ cells. Cluster 1 represents the start of pseudotime, with cluster 4 at the end. **d** Violin plots show the expression patterns of marker genes during oogenesis. **e** Hierarchical clustering of the ordering genes during early oogenesis. Each row represents an ordering gene, and each column represents a single germ cell. The GO terms of clustered ordering genes are listed on the right.

To explore the FGC development at a higher resolution, we isolated and re-clustered the germ cell populations, which were visualized using UMAP (Fig. 2a, b). To further recapitulate the trajectory of early FGC development, we constructed the germ cell lineage using Monocle2 package^10^, which organized the cells along the pseudotime trajectory, with mitotic FGCs and oogenesis phase FGCs concentrated at the beginning and end of its axis, respectively (Fig. 2c).

The reconstruction of the germ cell lineage divided the germ cell lineage into three developmental windows defined by the branch points of the pseudotime trajectories (Fig. 2c), allowing us to identify transition states leading to the oogenesis. We found that two waves of oogenesis occur in the germ cell lineage at the branch points 1 and 2. Noticeably, the first wave of oogenesis (branch 1) occurs following the RA-responsive stage, whereas the second wave of oogenesis (branch 2) occurs in the late meiotic prophase (Fig. 2c). The first wave of oogenesis may contribute to the medullary follicles that are activated immediately after their assembly, while the second wave of oogenesis contributes to the cortical primordial follicles that are activated after puberty and recruited regularly under hormonal control^11,12^. To further determine the patterns of differentially expressed genes, we examined the stage-specific marker genes, such as *SALL4*, *LIN28A*, *REC8*, *MSX1*, *SYCP1*/*2*, and *JAG1*, which change dynamically over the trajectory and are involved in oogonia proliferation, meiosis initiation, meiosis progression, and primordial follicle formation, respectively (Fig. 2d and Supplementary Fig. S1b). Also, to dissect the features of various stages of early FGC development, 1991 ordering genes that expressed dynamically along pseudotime were selected and clustered. The heatmap revealed stage-specific gene expression patterns that were consistent with well-organized germline development (Fig. 2e). As expected, gene ontology (GO) analysis of the clustered ordering genes (Supplementary Data S1) identified several significantly enriched biological processes, such as “mitotic cell cycle process” and “meiosis cell cycle process”. In summary, these data displayed the dramatic changes of transcriptomes during early germ cell development.

### Dynamic transcription factors control meiosis initiation and progression

**Fig. 3:**
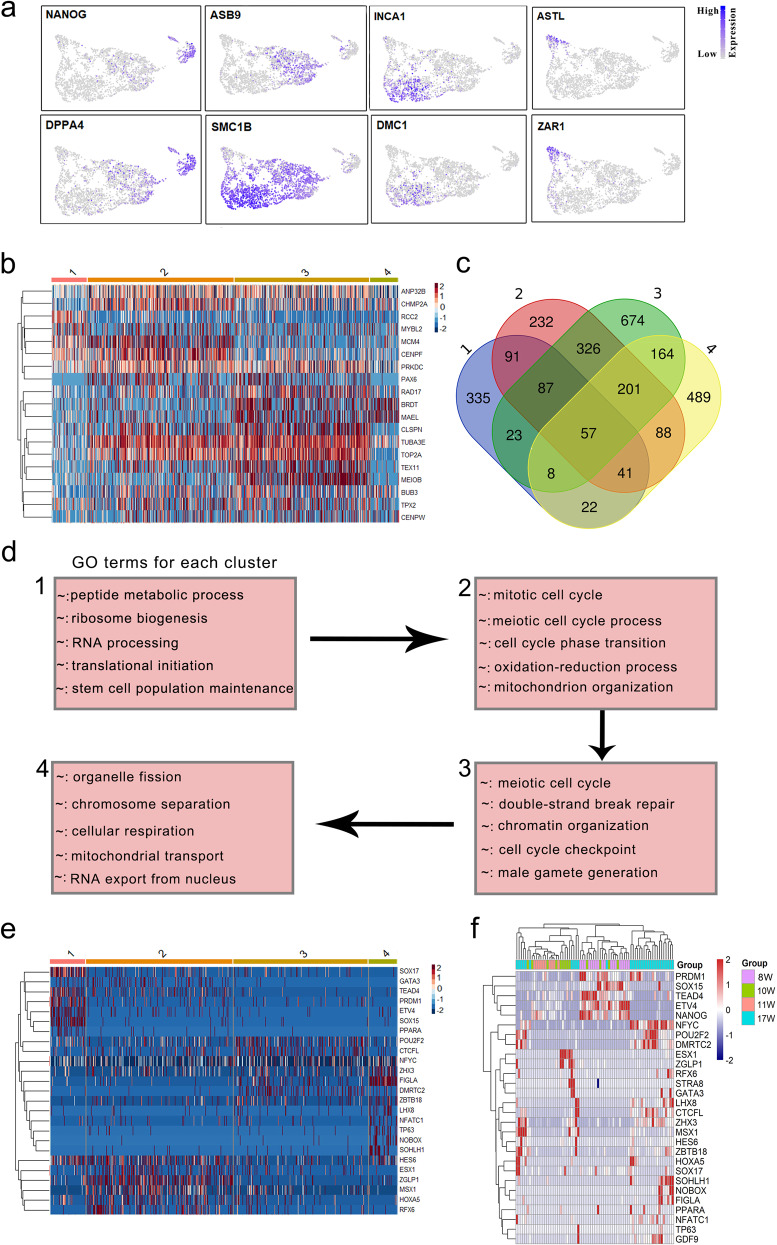
Mitosis to meiosis transition and meiosis progression. **a** Expression patterns of germ cell development-associated genes. **b** Hierarchical clustering of differentially expressed mitotic and meiotic genes across female germ cell clusters (clusters 1–4). **c** Overlap of differentially expressed genes across germ cell clusters. **d** Differentially expressed genes and associated GO categories characteristic of meiosis initiation and progression, based on the four germ cell clusters. **e** Heatmap representing the key transcription factors across monkey germ cell clusters. **f** Hierarchical clustering of the transcription factors and human female germ cells. Each row represents a transcription factor, and each column represents a single human female germ cell.

Having identified the cell clusters and constructed the developmental trajectory of FGCs, we next determined the transcriptome of FGCs during their development. Notably, mitotic phase FGCs clearly expressed pluripotent markers such as *NANOG* and *DPPA4*^13,14^ (Fig. 3a). Subsequently, the total expression levels of pluripotent genes significantly decreased during the transition from mitosis to meiosis, while meiotic marker genes, such as *DMC1* and *INCA1*, increased strikingly (Fig. 3a). In oogenesis phase FGCs, maternal effect genes, such as *ASTL* and *ZAR1*, began to express (Fig. 3a), suggesting the start of primordial follicle formation and folliculogenesis. To further explore the transition from mitosis to meiosis, we clustered the mitotic and meiotic genes across germ cell populations. As expected, mitotic genes, such as *RCC2* and *MYBL2*, were mainly expressed in cluster 1, while meiotic genes, such as *TEX11* and *MEIOB*, were mainly expressed in cluster 3 (Fig. 3b). Also, the *CENPF*, *PRKDC,* and *CHMP2A* exhibited peak expression in RA-responsive FGCs (Fig. 3b), which may play important roles in meiosis entry.

To further dissect the differences in gene expression profiles, we detected 664, 1123, 1540, and 1070 differentially expressed genes (Supplementary Data S2) among the first four clusters, respectively. And these differentially expressed genes which changed significantly during the mitosis to meiosis transition and meiosis to oogenesis transition (Figs. 1e and 3c). Also, we performed GO analysis on the differentially expressed genes in each of the germ cell clusters (Supplementary Data S3). The genes in cluster 1 were enriched in the GO terms of “peptide metabolic process” and “stem cell population maintenance”, while the genes in cluster 3 were enriched in the categories of “meiotic cell cycle” and “chromatin organization involved in meiosis”, which showed striking meiosis-related features (Fig. 3d). Noticeably, RA-responsive phase FGC-specific genes were enriched in mitotic and meiotic cell cycle processes, suggesting that the cell cycle phase transition occurs at this stage.

To further explore the regulation of meiosis initiation and progression, we analyzed the differentially expressed transcription factors across germ cell clusters (Fig. 3e). We found that transcription factor genes like *TEAD4*, *ETV4*, *PRDM1*, *SOX15*, and *SOX17* showed expression peaks specifically in the mitotic stage FGCs, which may be involved in primordial germ cell proliferation. Additionally, *ESX1*, *MSX1*, *RFX6*, and *ZGLP1* exhibited peak expression in RA-responsive stage FGCs. Evidence has shown that *MSX1* and *ZGLP1* are associated with meiosis initiation^15,16^, but no reports exist yet about the functions of *ESX1* and *RFX6* in meiosis. Noticeably, *DMRTC2* was specifically expressed in cluster 3 (Fig. 3e and Supplementary Fig. S1c), which might play an important role in meiosis progression^17^. For cluster 4, cells showed high levels of folliculogenesis-associated genes such as *FIGLA*, *NOBOX*, and *SOHLH1*. To further compare the differences between human and monkey FGC development, we downloaded the scRNA-seq data of human FGCs^5^, and analyzed the expression patterns of these transcription factors (Fig. 3f). As expected, the majority of transcription factors exhibited similar patterns between human and monkey FGCs. Noticeably, both *SOX15* and *SOX17* were highly expressed in monkey mitotic FGCs, while only *SOX15*was highly expressed in human mitotic FGCs (Fig. 3e, f). Moreover, hierarchical cluster analysis of these transcription factors also showed that there was a slight difference in the expression patterns of transcription factors between human and monkey FGCs (Fig. 3e, f). In addition, the zinc-finger protein family plays important roles in early oogenesis. For example, *ZNF625*, *ZNF217*, and *ZNF728* were mainly expressed in cluster 1, while *ZNF131* and *ZNF711* exhibited peak expression in cluster 3 (Supplementary Fig. S1d). Overall, our data demonstrate that the different germ cell clusters may represent different cell stages en route to early oogenesis.

### Majority of FGCs adopt an apoptotic fate

**Fig. 4:**
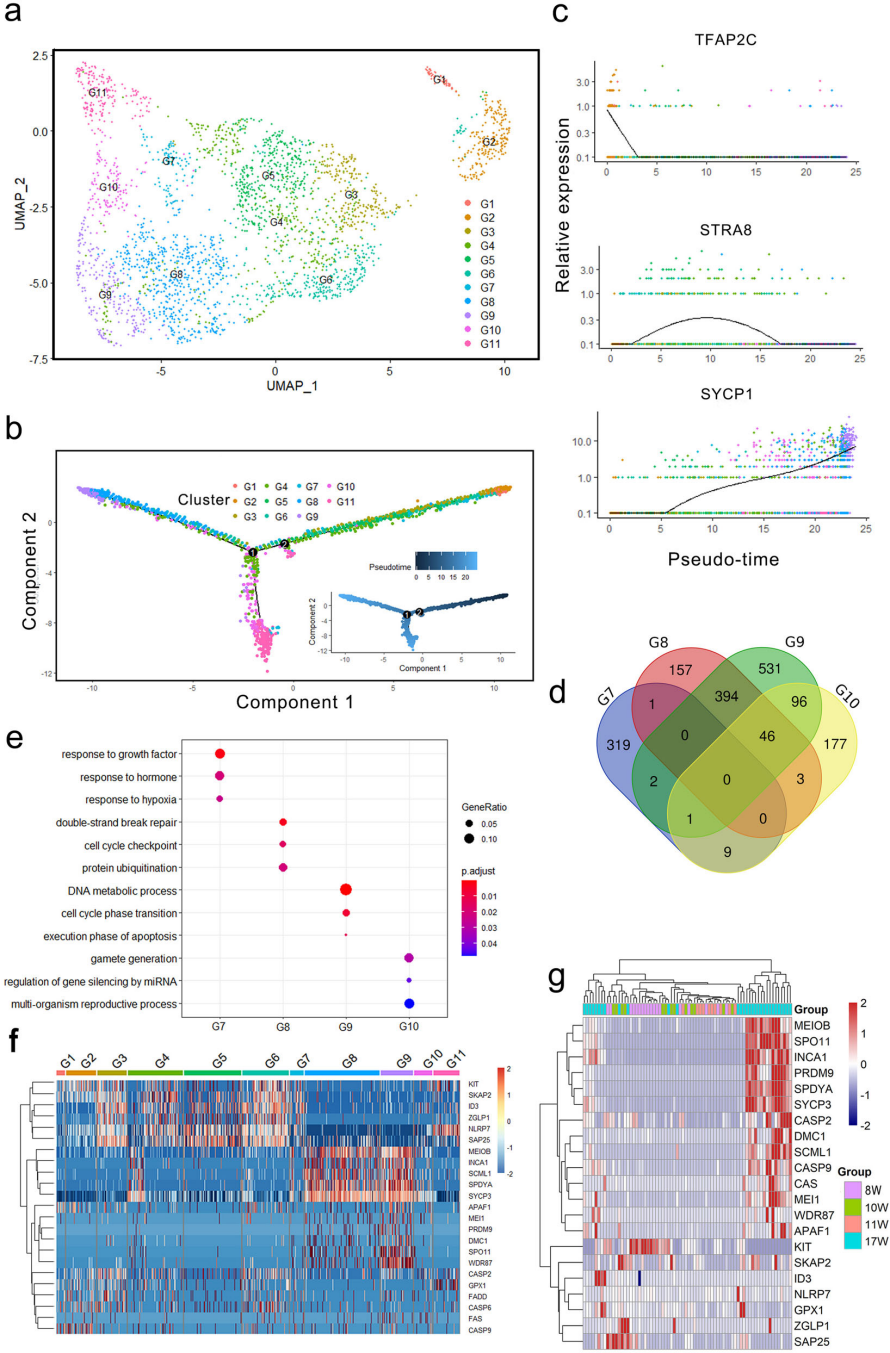
Female germ cell fate determination. **a** Re-clustering of germ cells at a higher resolution. The 11 sub-clusters (G1–G11) were assigned and colored as indicated on the figure key. **b** Cell lineage reconstruction using monocle2. Sub-cluster G1 represents the start of pseudotime, with sub-cluster G11 at the end. **c** Dynamic expression of stage-specific genes along pseudotime. **d** Overlap of highly variable genes among G7–G10 sub-clusters. **e** GO analysis of differentially expressed genes in clusters G7–G10. **f** Heatmap representing the survival, apoptosis, and meiotic genes in monkey female germ cells. **g** Heatmap representing the survival, apoptosis, and meiotic genes in human female germ cells. Each row represents a gene, and each column represents a single human female germ cell.

In contrast to the second wave of oogenesis, the majority of FGCs may undergo apoptosis after the branch point 2 (Fig. 2c). To dissect the mechanisms of germ cell fate determination, we performed re-clustering of the germ cells at a higher resolution and reconstructed the germ cell lineage using Monocle2. As a result, 11 sub-clusters were identified and the G8 and G9 may likely undergo apoptosis, while the G7 and G10 may generate primordial follicles (Fig. 4a, b). After entering meiosis, primordial germ cell-associated genes, such as *TFAP2C*, were down-regulated, while meiotic genes, such as *STRA8*, were up-regulated (Fig. 4c). Notably, the G8 and G9 at the end of the pseudotime exhibited sharply increased expression of meiotic genes, such as *SYCP1* and *SYCP2*, while G7 and G10 highly expressed *MT1X* that is essential for anti-apoptosis (Fig. 4c and Supplementary Fig. S2a)^18^. To identify genes that were exclusively expressed in a given cell type, we performed differential gene expression analysis to identify highly variable genes for each cluster (Supplementary Data S4). Venn diagram shows the significant differences between G7/G10 and G8/G9 (Fig. 4d). Moreover, GO analysis on the differentially expressed genes across from G7 to G10 showed that the GO term “execution phase of apoptosis” was highly enriched in the G9 cluster (Fig. 4e), suggesting that the cells in G9 may undergo apoptosis.

To further dissect the differences between apoptotic and oogenic germ cells, we selected several marker genes that were differentially expressed among these clusters (Fig. 4f and Supplementary Fig. S2b). For example, *NLRP7* is specifically expressed in germ cells that undergo oogenesis^19^, but this gene is not expressed in G8 and G9, which indicates that the cells in cluster G8 and G9 are probably primed for the apoptosis process. Moreover, several genes associated with germ cell development, such as *KIT* that is essential for germ cell survival^20^, also exhibit similar expression patterns with *NLRP7*. Furthermore, *ZGLP1* was shown to induce the oogenic fate in mice^16^, which is absent in clusters G8 and G9 but expressed in other meiotic germ cells. In contrast, meiotic genes, such as *SPO11*, *PRDM9*, and *DMC1*, were specifically expressed in G8 and G9 clusters. And PRDM9-mediated H3K4me3 at hotspots could direct double-strand breaks (DSBs) fate during meiosis recombination^21^. Moreover, *WDR87*, *SPDYA*, and *SCML1* also exhibit higher expression in G8 and G9, which may be critical for the regulation of germ cell fates (Supplementary Fig. S2b). In addition, hierarchical cluster analysis of these genes also showed that the apoptosis-associated genes, such as *CSAP2*, *CASP9,* and *APAF1*, were close to the meiotic genes, such as *PRDM9*, *DMC1*, and *SPO11*, rather than *NLRP7* and *KIT*. Furthermore, *CSAP2*, *CASP9*, and *APAF1* exhibited similar expression patterns with *PRDM9*, *DMC1*, and *SPO11* in human FGCs (Fig. 4g), which further confirms that the germ cells that sharply express meiotic genes, such as *SPO11*, *DMC1*, and *PRDM9*, may undergo apoptosis. Collectively, our findings indicate that the majority of FGCs are probably ready to undergo apoptosis, which may contribute to FGC attrition^22^.

### Identification of the granulosa cell subpopulations

**Fig. 5:**
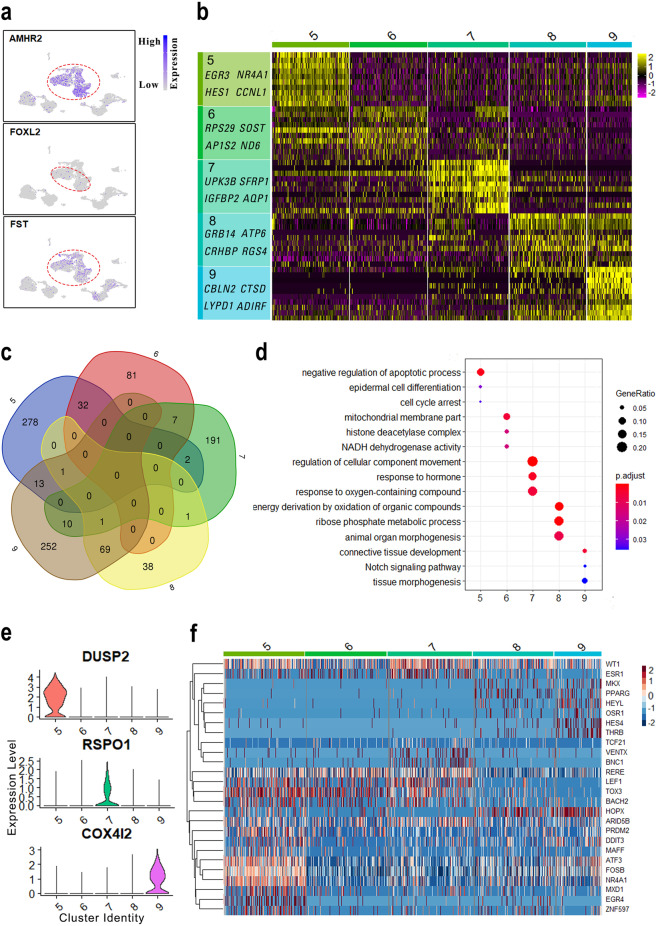
Dynamic changes of granulosa cell transcriptomes. **a** The expression of granulosa cell marker genes, with their expression projected onto the UMAP plot. **b** Heatmap showing representative marker genes of granulosa cell subpopulations (clusters 5–9). **c** Venn diagram shows overlapping of differentially expressed genes among granulosa cell populations. **d** The enriched GO terms (biological processes) to the subpopulations of the granulosa cells (clusters 5–9). **e** Violin plots show the specific expressed genes in different granulosa cell subpopulations. **f** Heatmap representing the dynamics of transcription factors across granulosa cell populations.

Granulosa cells are the most important somatic cells in the ovary, which markedly express *AMHR2*, *FOXL2*, and *FST* genes (Fig. 5a), and play important roles in FGC development and in the formation of primordial follicles. To examine the features of granulosa cells more closely, we detected the differentially expressed genes in each cluster, and the highly variable expressed genes in granulosa cells were identified (Fig. 5b). For example, *HES1*, *NR4A1*, and *EGR3* were mainly expressed in cluster 5, while *ADIRF*, *CBLN2*, and *LYPD1* exhibited peak expression in cluster 9. Subsequently, we performed principal component analysis (PCA) on the significant principal components to compare the transcriptomes of granulosa cells (Supplementary Fig. S3a). The PC1 axis had the most differences between the two samples. The E84 sample was located on the left side, whereas the E116 sample was located on the right side. The PC2 axis was dominated by differences in the cluster level within granulosa cells. However, we did not find any distinct boundary to group the granulosa cells.

To further explore the differences among granulosa cell clusters, we detected 326, 120, 212, 110, and 346 genes that differentially expressed across granulosa cell populations. Venn diagram shows that there are significant differences between cluster 5 and cluster 8 as well as cluster 6 and cluster 9 (Fig. 5c). This result suggests that the granulosa cells have undergone dramatic changes from E84 to E116 stages. To ascertain the features of granulosa cell subpopulations, we performed GO analysis on the differentially expressed genes in each cluster (Fig. 5d). As a result, the genes in cluster 5 were enriched in the categories of “negative regulation of apoptotic process” and “cell cycle arrest”, while the genes in cluster 9 were enriched in the GO terms of “notch signaling pathway” and “tissue morphogenesis”. Noticeably, certain genes in cluster 7 were enriched in the categories of “response to hormone”. Collectively, these results indicated that subpopulations of granulosa cells play distinct roles in fetal ovary development.

To dissect the mechanisms that regulate granulosa cell development, we analyzed the differentially expressed genes and transcription factors across granulosa cell clusters. We found that highly variable genes like *RSPO1*, *DUSP2*, and *COX4I2* exhibited peak expression specifically in the cluster 5, cluster 7, and cluster 9, respectively (Fig. 5e). Noticeably, *RSPO1*, an activator of the WNT/β-catenin pathway, is located upstream of the female sex determination signaling pathway, which could promote cell proliferation and ovarian differentiation^23,24^. In contrast, *DUSP2* is a substrate for mitogen-activated protein kinases (MAPKs)^25^, which may play an important role in inhibiting cell proliferation. Additionally, *COX4I2* is specifically expressed in cluster 9, which is involved in the modulation of oxygen affinity through hypoxia-sensing pathways, suggesting its role in the activation of primordial follicles^26^. Interestingly, *RDH10* also expressed in cluster 9 (Supplementary Fig. S3b), which is essential for the production of RA^27^. On the other hand, transcription factors were clustered across granulosa cell subpopulations (Fig. 5f). The *EGR4*, *ZNF597*, *DDIT3*, *MXD1*, and *MAFF* specifically expressed in cluster 5, while *MKK*, *PPARG*, *OSR1*, *HES4*, *THRB*, and *HEYL* mainly expressed in cluster 8 and cluster 9. Additionally, *ATF3*, *FOSB*, and *NR4A1* displayed higher expression in cluster 5 than other populations, whereas *ESR1* exhibited peak expression in cluster 7, which is consistent with the “response to hormone” category in cluster 7. Collectively, highly variable expressed transcription factors may instruct the differentiation of granulosa cells in a distinct manner.

### Similar characteristics between progenitor theca cells and fetal Leydig cells

**Fig. 6:**
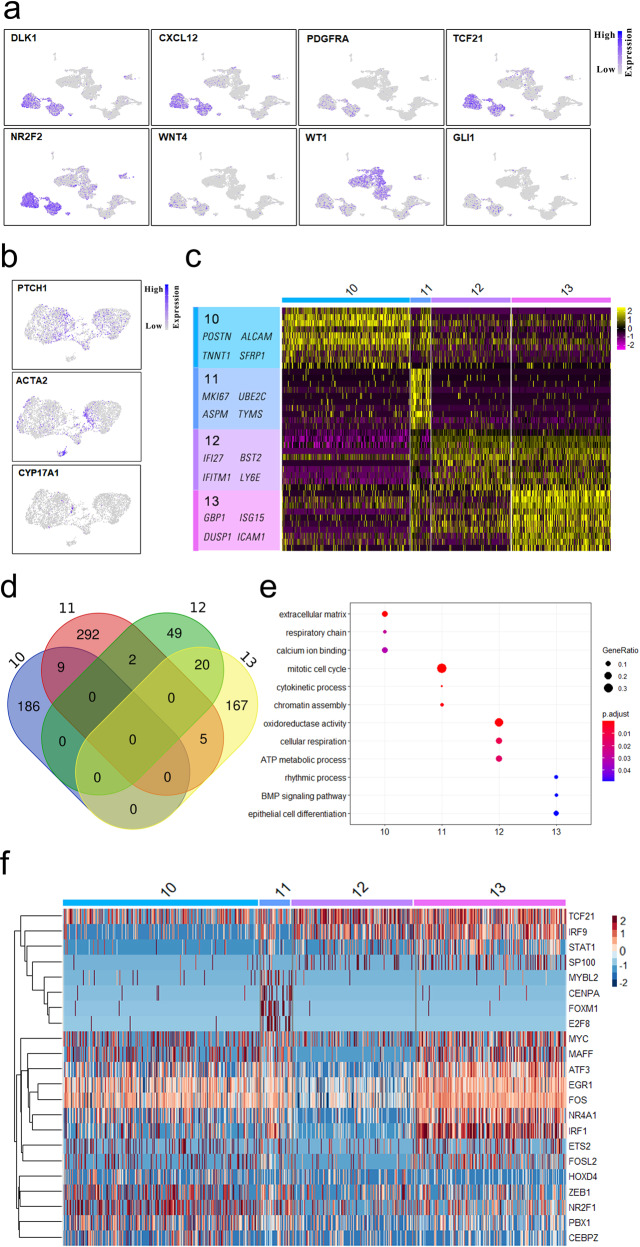
Dynamic changes of transcriptome in theca cells. **a** UMAP cluster map showing expression of selected known marker genes for Leydig cells. **b** The expression patterns of theca cell-associated marker genes. **c** Heatmap showing representative differentially expressed genes across theca cell populations. **d** Venn diagram shows overlapping of differentially expressed genes among theca cell populations. **e** Top GO terms within the differentially expressed genes unique to the theca cells. **f** Hierarchical clustering of the critical transcription factors among theca cell subpopulations.

Theca cells are another important somatic cell type in the ovary, which is required for folliculogenesis. However, the origin of theca cells has not been fully demonstrated. To explore the origin of theca cells, we performed scRNA-seq on the whole fetal ovary. Surprisingly, in addition to granulosa cells, there are four groups of cells that highly expressed fetal Leydig cell markers, such as *DLK1*, *CXCL12*, *PDGFRA*, and *TCF21* (Fig. 6a). Therefore, we named these cells Leydig cells. Noticeably, Leydig cells also express *NR2F2*, *WT1*, and *GLI1* (Fig. 6a), which suggested that Leydig cells may be the progenitor theca cells^2,28^. To further determine the origin of theca cells, we re-clustered the Leydig cells at a higher resolution and detected the levels of theca cell marker genes. As expected, the structural theca cell marker genes *PTCH1* and *ACTA2* as well as endocrine theca cell marker gene *CYP17A1* exhibited differentially expressed patterns across Leydig cell populations (Fig. 6b and Supplementary Fig. S3c), which indicated that progenitor theca cells exhibit the similar characteristics to fetal Leydig cells.

To further characterize theca cells, we detected the highly variable expressed genes across theca cell populations (Fig. 6c and Supplementary Fig. S4a). Obviously, cluster 11 is significantly different from the other three clusters. For example, *MKI67*, *SGO1*, *CDK1*, *CCNA2*, and *CENPF* were specifically expressed in cluster 11, whereas *IFI27*, *IFITM1*, and *BST2* were generally expressed in cluster 12 and cluster 13 (Fig. 6c and Supplementary Fig. S4a). Additionally, 195, 308, 71, and 192 differentially expressed genes were detected across theca cell populations. Similar to granulosa cells, theca cells exhibited distinct features between each cluster (Fig. 6d). To explore the features of theca cells, we carried out GO analysis on the differentially expressed genes in each cluster (Fig. 6e). Noticeably, the genes in cluster 13 were enriched in GO terms of “rhythmic process” and “BMP (bone morphogenic protein) singling pathway”, which suggests that theca cells in cluster 13 may be involved in folliculogenesis. To dissect the mechanisms that control theca cell differentiation, we clustered the transcription factors across theca cell populations. Obviously, the transcription factors in cluster 11, such as *MYBL2*, *CENPA*, *FOXM1*, and *E2F8*, displayed significant differences compared to that in the other three theca cell clusters (Fig. 6f). The fate of cells in cluster 11 needs to be further explored.

### Signaling pathways and interactions between niche and germ cells throughout fetal ovary development

The mitosis to meiosis transition and primordial follicle formation processes are crucial steps during fetal ovary development in primates. However, the molecular mechanisms and signaling pathways that are involved in these processes have not been fully explored. Therefore, we investigated the changes in RNAs encoding signaling pathway-associated factors during fetal ovary development to provide insights into FGC development and niche-germ cell interactions.

**Fig. 7:**
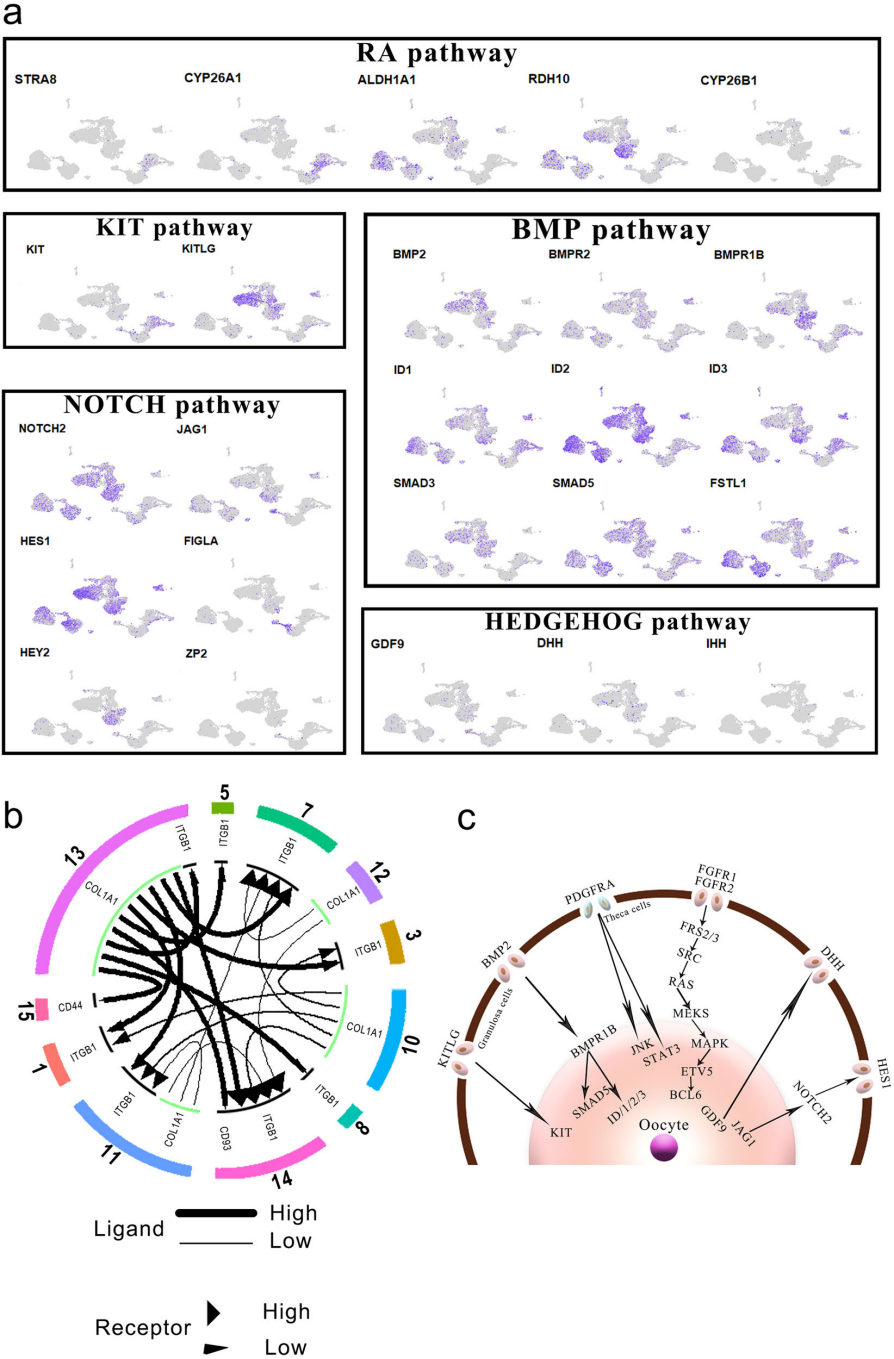
Signaling pathways for niche–germline interactions. **a** Relative expression levels of marker genes from different key signaling pathways. **b** Cell–cell communication networks between different cell clusters. **c** Schematic summary of signaling pathways for niche–germline interactions.

Our sequencing data showed that the RA signaling pathway and BMP signaling pathway are involved in oogenic program initiation and meiotic progression. For the BMP signaling pathway, the ligand *BMP2* was highly expressed in granulosa cells, whereas the receptor *BMPR1B* was expressed in both granulosa cells and FGCs. Noticeably, the targets *ID1*, *ID2*, and *ID3* were expressed in FGCs in a stage-specific manner (Fig. 7a). This pattern suggests that the BMP signaling pathway may play an important role in meiosis initiation and progression. Additionally, the ligand of the KIT signaling pathway (*KITLG*) was specifically expressed in granulosa cells, and the receptor *KIT* was highly expressed in FGCs, which is crucial for germ cell survival^20^. Interestingly, *PDGFB* was specifically expressed in endothelial cells, and its receptors *PDGFRA* and *PDGFRB* were found in theca cells and partial granulosa cells (Supplementary Fig. S4b), suggesting that endothelial cells may indirectly affect FGC development, through the interactions with other niche cells. To further explore the crosstalk among different cell clusters within the monkey fetal ovary, we established the cell–cell communication network using iTALK package^29^. Notably, nearly all the ligands were derived from progenitor theca cells, and the associated receptors were enriched in other cell types (Fig. 7b), which suggests the important roles of theca cells in ovary development.

To dissect the interactions between the oocyte and granulosa cells throughout folliculogenesis, we further analyzed the expression of the factors in key signaling pathways, including NOTCH and HEDGEHOG pathways. Our results showed that the ligand *JAG1* of NOTCH pathway was predominantly expressed in the oocytes at the oogenesis phase, whereas the receptor *NOTCH2* as well as the downstream target gene *HES1* were highly expressed in granulosa cells and progenitor theca cells (Fig. 7a). Next, we investigated the key components of the HEDGEHOG signaling pathway in the oocytes and somatic cells. *GDF9* exhibited a high expression level in the oogenesis phase FGCs, which could promote the production of Desert hedgehog and Indian hedgehog in granulosa cells. Moreover, the receptor *PTCH1* as well as downstream signaling components *GLI1* of the HEDGEHOG pathway were highly expressed in theca cells (Fig. 7a, c), indicating that the HEDGEHOG pathway may play critical roles in the specification of theca cells. Taken together, our results demonstrate that reciprocally interacting signaling pathways control fetal ovary development in a stage-specific manner.

## Discussion

Fetal ovary development is a complex process in primates, and a full understanding of its regulation requires the integration of multiple data collected from various cell types in the ovary. Here, we performed scRNA-seq analysis of all cells within the fetal ovaries at two different developmental stages to provide new insights into the regulation of fetal ovary development in primates. In this study, we have identified four clusters of FGCs: mitotic phase, RA-responsive phase, meiotic phase, and oogenesis phase. Moreover, the niche cells, such as granulosa cells, theca cells, endothelial cells, and macrophages, were also identified. Furthermore, our work revealed unique characteristics in transcriptional profiles and reciprocal interactions between germ cells and niche cells in each developmental stage. These identified cell types and stage-specific expressed genes in fetal ovaries may offer a valuable information for future functional studies.

FGCs undergo meiosis initiation, primordial follicle formation, and apoptosis. Here, we reconstructed the developmental trajectory of FGCs, which revealed two waves of oogenesis. Noticeably, the first minor oogenesis occurs following the RA-responsive stage, which could further undergo growth and development in the medulla^12^. However, these follicles have to undergo atresia due to lacking hormone stimulation in the prepuberty ovary, and cannot contribute to fertility. On the other hand, the second major oogenesis occurs in the late meiotic prophase accompanied with the apoptosis of the vast majority of germ cells. The germ cells in the second oogenesis will form primordial follicles and stay dormant in the ovarian cortex until puberty^11^. After puberty, the primordial follicles are recruited regularly to generate mature oocytes under the stimulation of gonadotropin hormones, which contributes to the life span of female reproduction.

The mitosis to meiosis transition is a critical step in the oogenic program; however, the mechanisms for the regulation of meiosis initiation have not been fully demonstrated. Previous studies have shown that RA induces the expression of *STRA8*, which could further switch on the meiotic genes, such as *SYCP3*, and repress the early PGC program to promote meiosis initiation. RA enhances this pathway through retinoic acid receptor (RAR)-mediated transcriptional regulation; however, after ablation of RAR-coding genes, the FGCs robustly expressed meiotic genes, such as *STRA8*, *REC8*, and *SYCP3*, showing that RARs are actually dispensable for meiosis initiation^30^. Moreover, recent studies indicate that *Zglp1* is partially overlapped with *Nanog* in mice, which is sufficient to induce the oogenic fate and meiosis entry, whereas RA augments the ZGLP1-activated oogenic program and meiosis initiation^16,31^. In our study, *ZGLP1* is mainly expressed in germ cells at the RA-responsive stage, which is not overlapped with *NANOG* in monkey FGCs, indicating that *ZGLP1* is essential for meiosis entry but not for activating the oogenic fate in primates. Moreover, *ZGLP1* is also expressed in the oogenesis phase germ cells, suggesting its roles in subsequent oogenesis.

During the second wave of oogenesis, the germ cells are surrounded by somatic cells to form primordial follicles, while the vast majority of germ cells undergo apoptosis. Reconstructed germ cell lineage revealed that C8 and C9 are likely to undergo apoptosis, while other germ cells maintain survival and undergo oogenesis. *NLRP7* is generally expressed in all stages of oogenesis, and *KIT* is crucial for the survival of germ cells^19,20^. However, *NLRP7* and *KIT* were not expressed in the C8 and C9. Noticeably, several meiosis-associated genes, such as *SPO11*, *PRDM9*, *DMC1*, and *INCA1*, exhibited peak expression in C8 and C9. Recent studies have shown that PRDM9-mediated H3K4me3 could guide SPO11 targeting to induce DSBs^32^. Moreover, earlier formed DSBs occupy more open chromatin and are much more competent to proceed to a crossover fate, whereas later formed DSBs are likely to proceed to a non-crossover fate^21^. In this study, *PRDM9* and *SPO11* specifically expressed in C8 and C9, whose DSBs are formed late causing their non-crossovers fate, which may be the cause for apoptosis of germ cells in C8 and C9.

Ovary and testis have the same developmental origin: the bipotential gonads that are composed of multipotent somatic progenitor cells. After sex determination, the somatic progenitor cells differentiate into Sertoli cells and Leydig cells in male gonads, or granulosa cells and theca cells in female gonads. Our data revealed five clusters of granulosa cells that displayed distinct features in late stage ovaries. In addition, we also provide several new insights into theca cell development, most importantly into the origin of theca cells. Apart from the somatic progenitor cells that differentiated into granulosa cells, the remaining somatic progenitor cells exhibited peak expression of *NR2F2*, which acquire a steroidogenic precursor fate by progressively expressing *CYP17A1*, *PTCH1*, and *ACTA2*. Interestingly, these cells also exhibited the Leydig cell features, which highly expressed Leydig cell marker genes, such as *DLK1*, *TCF21*, and *CXCL12*. In contrast to *SRY* that is essential for male sex determination, *NR2F2* has been shown to control female sex determination through eliminating Wolffian ducts in female embryos^33^. Moreover, *NR2F2*, a lineage-specific transcription factor, plays important roles in cell-type specification and cell fate maintenance^34^. Therefore, we speculated that progenitor theca cells exhibit similar features to fetal Leydig cells, and the features of Leydig cells are eliminated gradually through the development of the ovary dependent on *NR2F2*.

A major area of current interest involves niche–germline communications that coordinately and reciprocally regulate fetal ovary development, but knowledge on how the signaling pathways interact remains limited. In this study, we have identified many ligands and receptors that are derived from the reciprocal compartments. For example, we found that the BMP signaling pathway was activated in FGCs via granulosa cells-driven mechanisms. BMP2, the ligands for the BMP signaling pathway, was specifically expressed in granulosa cells, while its receptor, BMPR1B, and downstream effector, *ZGLP1*, were expressed in FGCs. Our data concur that the BMP–ZGLP1 pathway could also activate the oogenic program in primates. Moreover, the typical RA pathway contributes to oogenic program maturation and PGC program repression. In contrast, NOTCH and HEDGEHOG pathways were activated in niche cells. For instance, the ligand *JAG1* of the NOTCH pathway was specifically expressed in the oocytes, while its receptor *NOTCH2* as well as the downstream target gene *HES1* were highly expressed in granulosa cells and progenitor theca cells. In this study, we have identified several pathways that may govern the fetal ovary development in primates, which provides valuable information for the improvement of in vitro culture of gametes.

In summary, this work provides new insights into the crucial features of monkey fetal ovaries especially in the late embryonic stages. Our study paves the way for understanding the molecular regulation of fetal FGC development, including meiosis initiation, primordial follicle formation, and apoptosis of germ cells. It also lays a solid foundation for the theca cell origin identity. More importantly, the reciprocal relationship between the signaling pathways of FGCs and their niche cells will provide a valuable resource for further optimizing and improving the efficiency of germ cell differentiation in vitro.

## Materials and methods

### Animal ethics statements

Fetal female cynomolgus monkeys (*Macaca fascicularis*) were selected for this study. The use and care of animals complied with the guideline of the Animal Advisory Committee at the Institute of Neuroscience, Chinese Academy of Sciences. The ethics application entitled “Construction of cynomolgus monkey model based on somatic cell nuclear transfer” (#ION-2018002R01) was approved by the Institute of Neuroscience, Chinese Academy of Sciences.

### Sample collection

The ovary samples for scRNA-seq were from two fetal female *Macaca fascicularis* (embryonic days 84 and 116). For each single-cell sequencing experiment, two ovaries were washed twice in 1× PBS, and subjected to a standard digestion procedure through Tumor Dissociation Kit, human (MiltenyiBiotec #130-095-929). First, the ovaries were cut into small pieces of 2–4 mm, and transferred into the gentleMACS C Tube containing the enzyme mix (4.7 mL DMEM, 200 µL Enzyme H, 100 µL Enzyme R, and 25 µL Enzyme A). Then, the C tube was attached onto the sleeve of the gentleMACSdissociator. After termination of the program, the C tube was detached from the gentleMACSDissociator. The sample was incubated for 30 min at 37 °C under continuous rotation using the MACSmix Tube Rotator. Next, the C tube was attached onto the sleeve of the gentleMACSdissociator, and then subjected to a short centrifugation step to collect the sample material at the bottom of the tube. The sample was resuspended and the cell suspension was applied to a MACS SmartStrainer (70 µm) placed on a 50 mL tube and washed in a cell MACS SmartStrainer (70 µm) with 20 mL DMEM. Finally, the cell suspension was centrifuged at 300× *g* for 7 min, the supernatant was completely aspirated and cells were resuspended as required for further applications.

### Construction of single-cell RNA libraries and sequencing

Single-cell suspensions of ovary cells were captured on a 10× Chromium system (10× Genomics). Then, single-cell mRNA libraries were generated using the single-cell 3′ reagent V3 kits according to the manufacturer’s protocol. About 16,000 cells were added to each channel with a targeted cell recovery estimate of 8000 cells (10,000 for E84 monkey and 12,947 for E116 monkey). After generation of GEMs, reverse transcription reactions were barcoded using a unique molecular identifier (UMI) and cDNA libraries were then amplified by PCR with appropriate cycles. Subsequently, the amplified cDNA libraries were fragmented and then sequenced on an Illumina NovaSeq 6000 (Illumina, San Diego).

### ScRNA-seq data processing and analysis

The Cell Ranger software (version 3.0.2) was used to perform sample demultiplexing, reads mapping and barcode processing to generate a matrix of gene counts versus cells. Briefly, the raw BCL files generated by Illumina NovaSeq 6000 sequencing were demultiplexed into fastq files through the Cell Ranger *mkfastq* pipeline. Next, the fastq files were processed using the Cell Ranger *count* pipeline to map high-quality reads to the monkey reference genome (Macaca_fascicularis_5.0). Aligned reads were further filtered for valid cell barcodes and UMIs to produce a count matrix.

Then, count matrix was imported into the R package Seurat^8^ and quality control was performed to remove outlier cells and genes. Cells with 200–3000 detected genes were retained. Genes were retained in the data if they were expressed in ≥ 3 cells. After applying these quality control criteria, 12471 cells and 18446 genes remained for downstream analyses. Additional normalization was performed in Seurat on the filtered matrix to obtain the normalized count. Highly variable genes across single cells were identified and PCA was performed to reduce the dimensionality on the top 18 principal components. Then, cells were clustered at a resolution of 0.6 and visualized in two-dimensions using UMAP^9^.

### Pseudotime analysis of single-cell transcriptomes

Germ cell lineage trajectories were constructed according to the procedure recommended in the Monocle2 documentation (http://cole-trapnell-lab.github.io/monocle-release/docs)^10^. Cluster 1 (mitotic PGCs) was used as start, cluster 2 (meiotic PGCs) as middle, and cluster 4 (early activated oocytes) as the end of pseudotime. Briefly, the top differentially expressed genes were selected as “ordering genes” to recover lineage trajectories in Monocle2 using default parameters. After pseudotime time was determined, differentially expressed genes were clustered to verify the fidelity of lineage trajectories. Additionally, the expression patterns of key germ cell markers across pseudospace were visualized through the function of plot_genes_in_pseudotime in Monocle2.

### GO analysis

Enrichment analysis for highly variable genes detected per cluster was conducted using ClusterProfiler R package^35^. Symbol gene IDs were translated into Entre IDs through bitr function. The analysis of the enrichment of differentially expressed genes was performed and a corrected *P* value ≤ 0.05 was considered to indicate significant gene enrichment.

### Transcription factor analysis

To show the dynamics of transcription factors during FGC and somatic cell development, we downloaded all 1426 monkey transcription factors from Animal TFDB 3.0 (http://bioinfo.life.hust.edu.cn/AnimalTFDB). Then, the differentially expressed genes across 15 cell clusters and monkey transcription factors were intersected to obtain differentially expressed transcription factors, which were further visualized using heatmap.

### Cell–cell communication analysis

Cell–cell communication was analyzed using iTALK package^29^. The input data were Seurat object, and the ligand–receptor pairs were detected from top 50% highly expressed genes. The communication types mainly included growth factors, cytokines, and checkpoint. The network plot was visualized using LRPlot function.

### Transcriptome analysis of human FGCs

The scRNA-seq data were downloaded from NCBI Gene Expression Omnibus (GEO) database^5^, and the associated accession number was GSE63818. The gene expression matrix was established through merging the data of 72 single cells ranging from 8 to 17 weeks (Supplementary Data S5). The dynamics of transcription factors and critical genes during FGC development were visualized using heatmap with hierarchical clustering of columns and rows.

## Supplementary information

Supplementary Information

Supplementary Data S1

Supplementary Data S2

Supplementary Data S3

Supplementary Data S4

Supplementary Data S5

## Data Availability

The high-throughput sequencing data in this study have been deposited in the Gene Expression Omnibus (GEO) database under accession number GSE149629. To review GEO accession GSE149629: Go to https://www.ncbi.nlm.nih.gov/geo/query/acc.cgi?acc=GSE149629. Enter token exkjqgaibhyfhqt into the box.

## Supplementary information

**Supplementary Information** accompanies the paper at (10.1038/s41421-020-00219-0).
