# Supplementary material for: Single-cell RNA sequencing reveals regulation of fetal ovary development in the monkey (Macaca fascicularis)
Source: Cell Discov. 2020 Dec 29;6:97. doi: 10.1038/s41421-020-00219-0 (PMC7769980; doi:10.1038/s41421-020-00219-0)
Supplement: Supplementary file 1 — Supplementary Information [file 41421_2020_219_MOESM1_ESM.pdf]

a

Sample	E84	E116
Total reads	492996867	711714630
Number of cells	10676	10360
Mean reads per cell	46178	68698
Genes detect	19163	19317
Median genes per cell	1403	1788

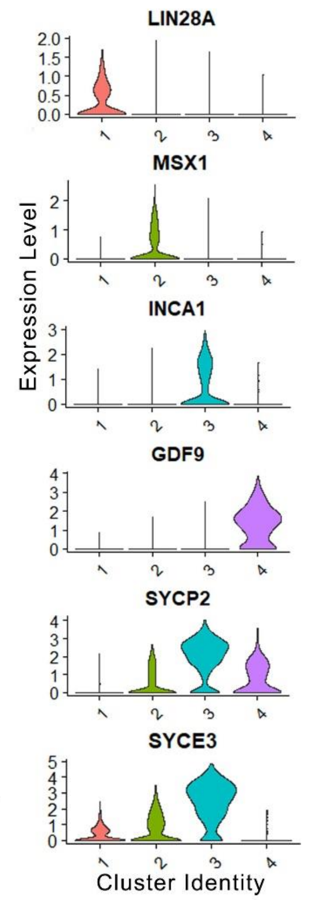

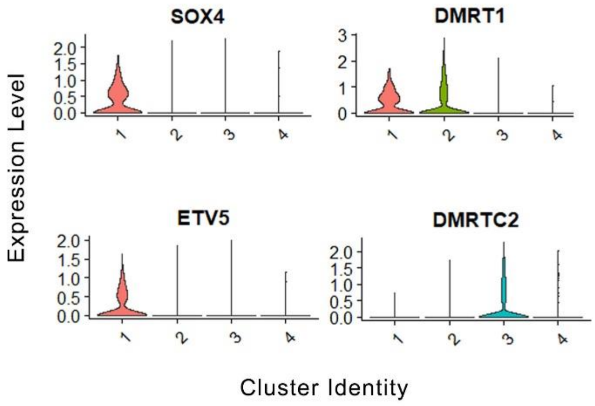

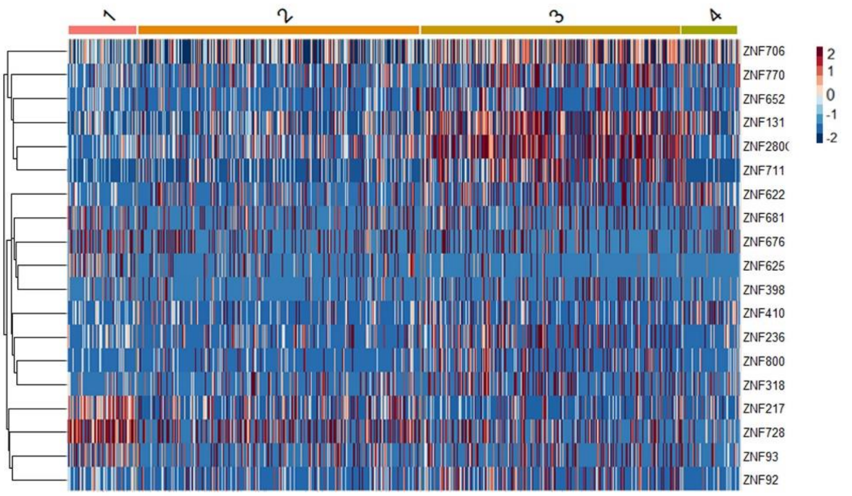

Supplementary Fig. S1 Sequencing information and differentially expressed genes in germ cell clusters. **a** Sequencing information including cell number, mean reads per cell and median genes per cell etc. **b** Violin plots show the expression patterns of marker genes across germ cell clusters. **c** Violin plots of the critical transcription factors across germ cell populations. **d** Heatmap representing the expression patterns of zinc finger protein family genes across germ cell clusters.

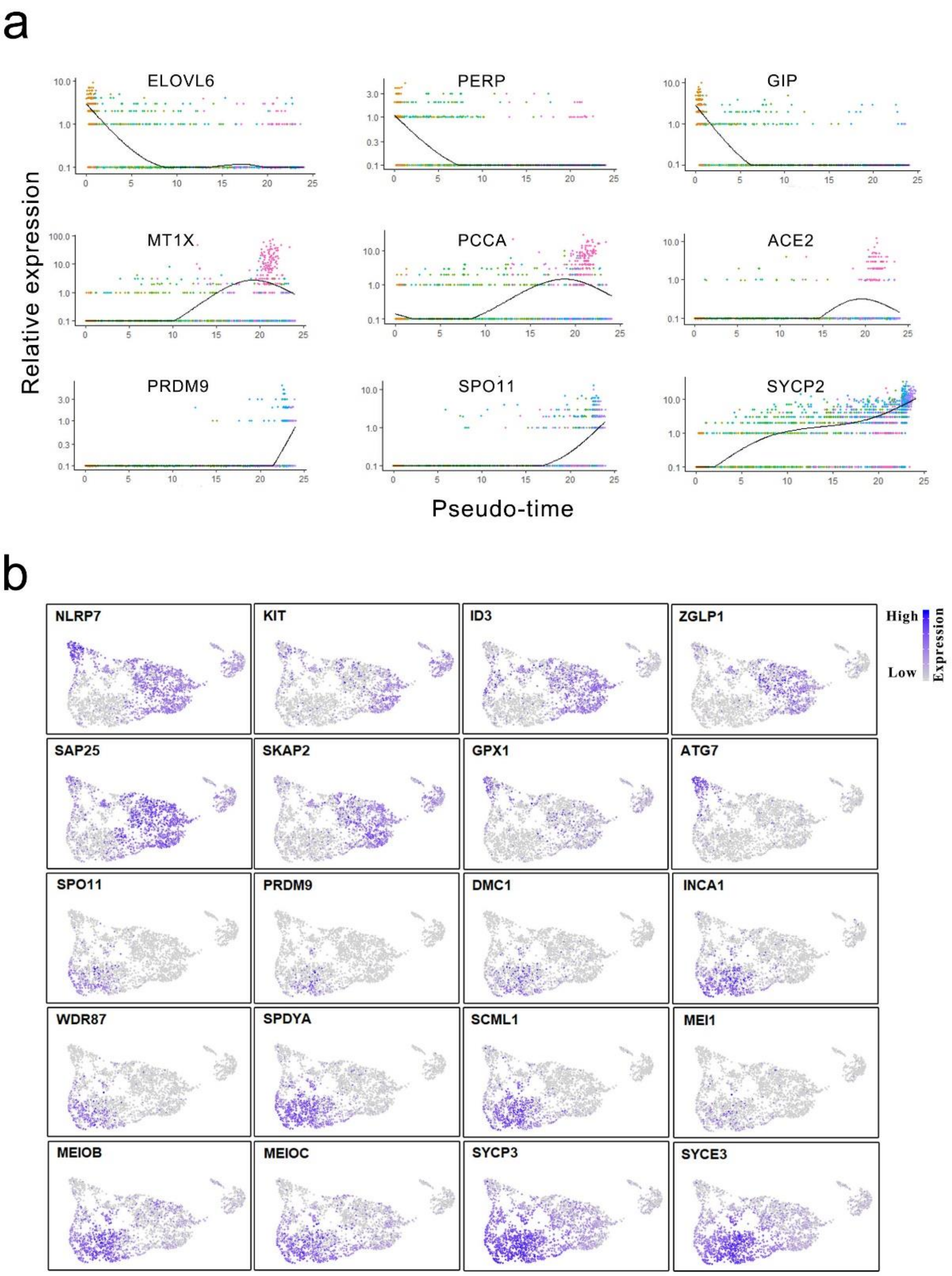

Supplementary Fig. S2 The expression patterns of highly variable genes in germ cell clusters. **a** Dynamic changes of highly variable genes along pseudo-time using monocle2. **b** The expression patterns of germ cell fate determination-associated genes.

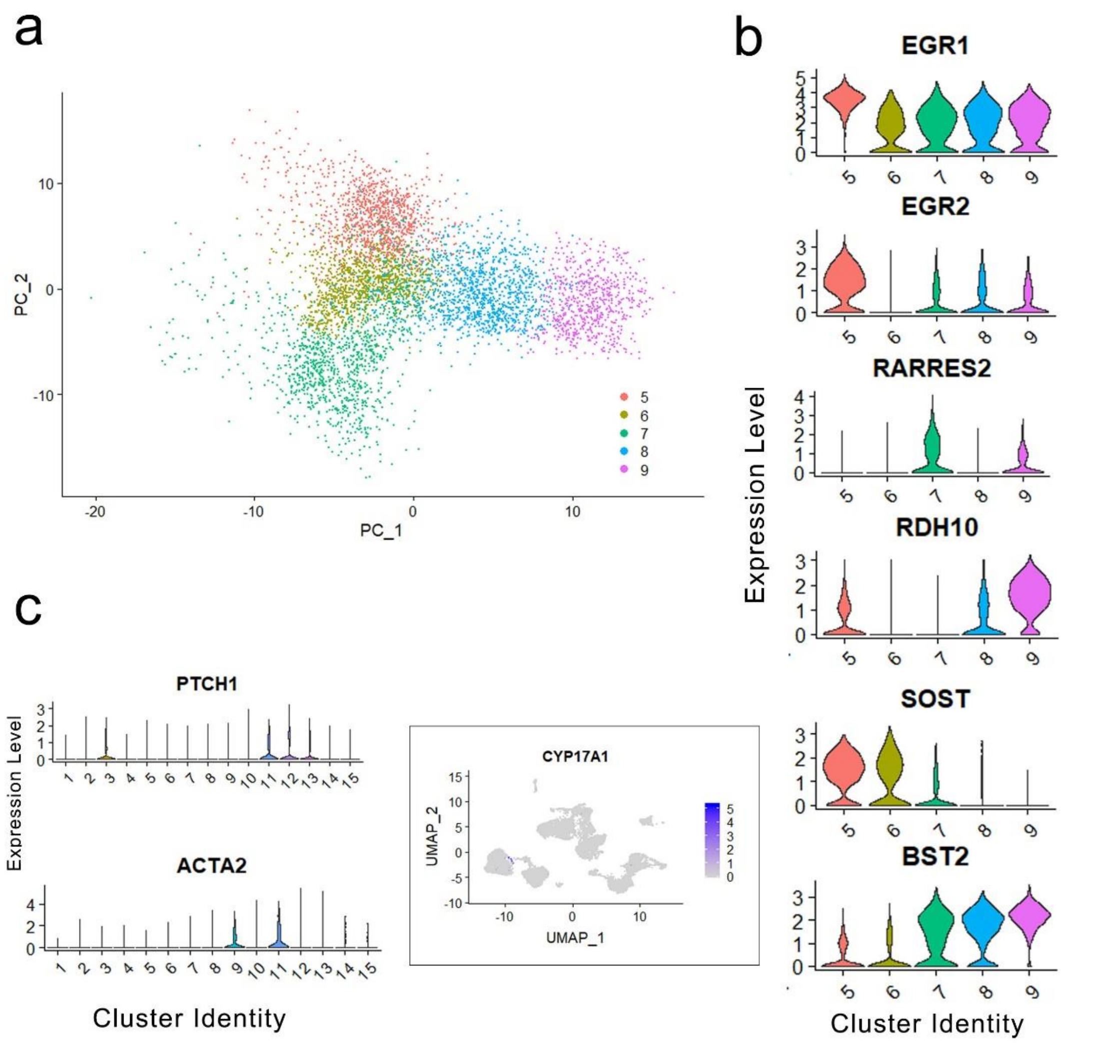

Supplementary Fig. S3 PCA analysis and gene expression patterns. a PCA analysis of the transcriptome of granulosa cells. **b** The expression patterns of highly variable genes among granulosa cell subpopulations. **c** The expression patterns of *PTCH1*, *ACTA2* and *CYP17A1*.

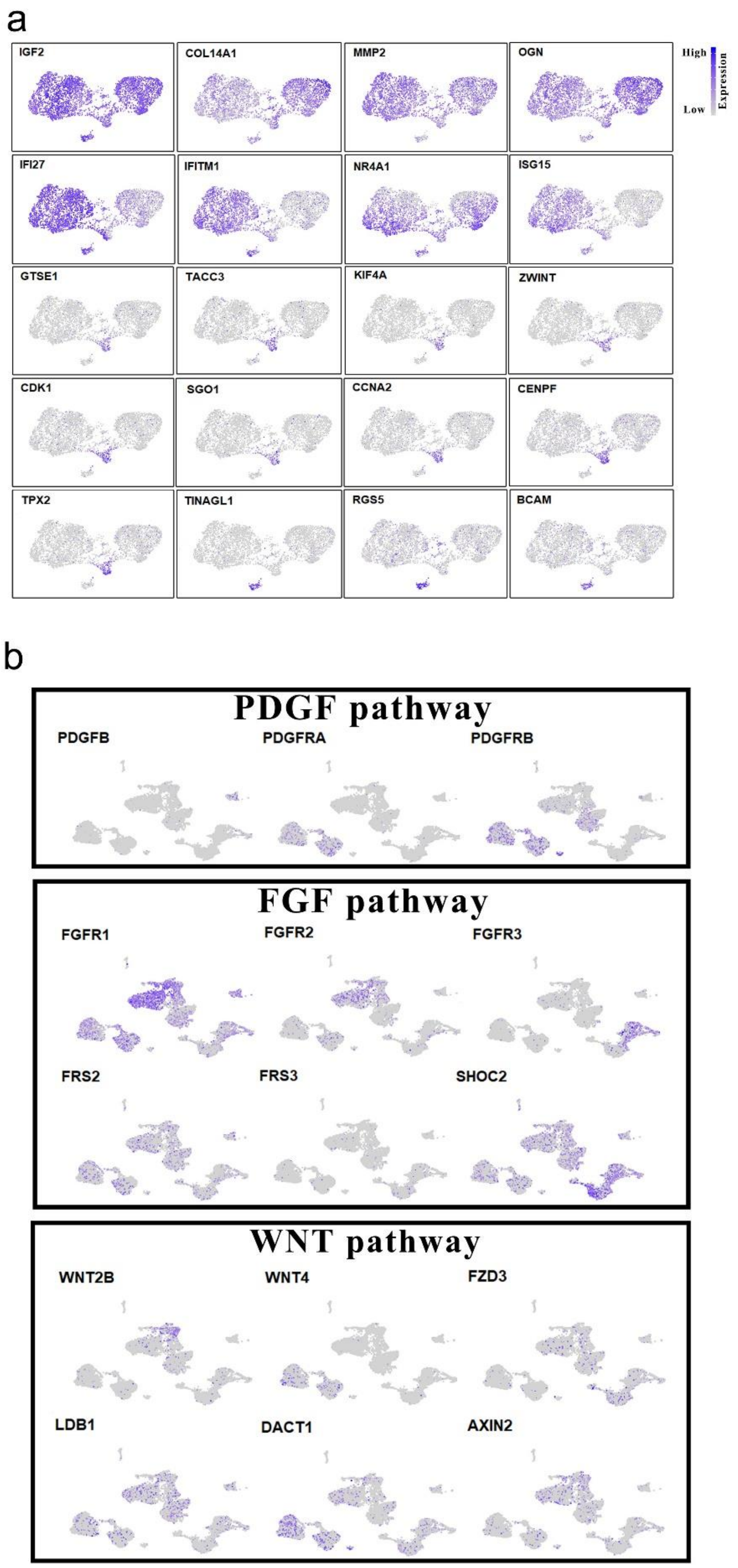

Supplementary Fig. S4 Differentially expressed genes and signaling pathways. a The expression patterns of highly variable genes among theca cell subpopulations. **b** The critical marker genes for different key signaling pathways.
